# Effect of Growth Media on the Diversity of *Neocallimastigomycetes* from Non-Rumen Habitats

**DOI:** 10.3390/microorganisms10101972

**Published:** 2022-10-05

**Authors:** Akshay Joshi, Diana Young, Liren Huang, Lona Mosberger, Bernhard Munk, Julia Vinzelj, Veronika Flad, Alexander Sczyrba, Gareth W. Griffith, Sabine Marie Podmirseg, Rolf Warthmann, Michael Lebuhn, Heribert Insam

**Affiliations:** 1 Biocatalysis, Environment and Process Technology Unit, Life Science and Facility Management, Zurich University of Applied Sciences (ZHAW), 8820 Wadenswil, Switzerland; 2 Department of Microbiology, University of Innsbruck, Technikerstrasse 25d, A-6020 Innsbruck, Austria; 3 Central Department for Quality Assurance and Analytics, Micro- and Molecular Biology, Bavarian State Research Center for Agriculture, Lange Point 6, 85354 Freising, Germany; 4 Faculty of Technology, Bielefeld University, 33615 Bielefeld, Germany; 5 Chair of Urban Water Systems Engineering, Technical University of Munich (TUM), 85748 Garching, Germany; 6 Department of Life Sciences, Cledwyn Building, Aberystwyth University, Aberystwyth SY23 3DD, UK

**Keywords:** anaerobic fungi, *Neocallimastigomycota*, ruminants, hindgut animals, minimal medium, novel genus, selective isolation, enrichment

## Abstract

Anaerobic fungi (AF), belonging to the phylum *Neocallimastigomycota,* are a pivotal component of the digestive tract microbiome of various herbivorous animals. In the last decade, the diversity of AF has rapidly expanded due to the exploration of numerous (novel) habitats. Studies aiming at understanding the role of AF require robust and reliable isolation and cultivation techniques, many of which remained unchanged for decades. Using amplicon sequencing, we compared three different media: medium with rumen fluid (RF), depleted rumen fluid (DRF), and no rumen fluid (NRF) to enrich the AF from the feces of yak, as a rumen control; and Przewalski’s horse, llama, guanaco, and elephant, as a non-rumen habitats. The results revealed the selective enrichment of *Piromyces* and *Neocallimastix* from the feces of elephant and llama, respectively, in the RF medium. Similarly, the enrichment culture in DRF medium explicitly manifested *Piromyces*-related sequences from elephant feces. Five new clades (MM1-5) were defined from llama, guanaco, yak, and elephant feces that could as well be enriched from llama and elephant samples using non-conventional DRF and NRF media. This study presents evidence for the selective enrichment of certain genera in medium with RF and DRF from rumen as well as from non-rumen samples. NRF medium is suggested for the isolation of AF from non-rumen environments.

## 1. Introduction

Anaerobic fungi (AF) play a vital role in the degradation of ingested forage in the gut of ruminants, hindgut animals, and other mammalian herbivores [1]. For several decades, the taxonomy of AF was mainly based on six genera—namely, *Neocallimastix*, *Caecomyces*, *Orpinomyces*, *Piromyces*, *Anaeromyces,* and *Cyllamyces* [2,3]. Recently, the diversity of AF is gaining a new shape—a total of 20 genera have been named within the phylum *Neocallimastigomycota*, comprising 36 species, of which half of the genera were described in the last five years [4,5], and many more still need to be discovered [1,6,7,8]. Several reasons explain the remaining gap of knowledge on AF diversity and their propagation, most prominently the relatively short history of AF isolation, improper sampling and isolation techniques, and insufficient sampling sources [9].

Since their discovery in 1975 until sequence-based identification techniques became widely available, AF were characterized only based on their morphological features [3,10,11]. Even if this is still essential for the description of novel AF, it involves insecurities due to the substrate-based polymorphism occurring in AF, hence, it resulted in the misclassification of novel isolates under the already-described genera [5,12]. An example for such a confounding classification, which was not clear based on morphology, was the description of the genus *Oontomyces*. The respective isolates showed morphological similarity with the monocentric genus *Piromyces* spp. but were genetically more closely related to the polycentric genus *Anaeromyces*. Furthermore, a more detailed morphological analysis following phylogenetic reconstruction can reveal hitherto unnoticed and distinctive morphological features [13], as was the case with *Pecoramyces ruminantium*, originally classified as *Orpinomyces* sp. [14].

Recently, with advances in molecular identification techniques, the existence of AF was also recorded in a broader range of host animals, including the feces of herbivorous reptiles and gorilla [6,15,16]. Further, a culture-independent diversity study suggested the existence of 34 uncultured AF genera and 119 species [17]. Such information is crucial to further decipher the ecology of AF and can be used as a map for the targeted isolation of AF. In addition, studies covering molecular identification, the selection of a marker gene, and specific primer pairs for discovering the utmost diversity of yet uncultivable AF are contradictory and difficult to compare. Most of these studies are based on the ITS (internal transcribed spacer) region as a marker [18]. Recently, Hanafy and collaborators analyzed the D1/D2 of the LSU (large ribosomal RNA gene) to explore all currently identified and uncultured genera of AF [7].

The rumen of fistulated or deceased animals was the most commonly sampled habitat for the isolation of AF in early studies [3,19,20,21]. Later, fresh and air-dried feces were also used for isolation [22]. Most of the presently known genera were initially isolated from the digesta and feces of small and large ruminants, except *Caecomyces* and *Khoyollomyces,* which were isolated from the feces of horse [5,10,23]. Enrichment and isolation medium for AF was originally developed to mimic the environmental and nutritional conditions in the rumen and it has remained largely unchanged since the first isolation of AF in 1975 [3]. Culture media, which are to date routinely applied to enrich AF, are non-defined and complex media containing clarified rumen fluid (RF) in a concentration of about 15% [22,24,25]. As the isolation sources of AF have notably changed, and now include non-rumen environments, the rumen-based media may be suboptimal for the latter and distort the diversity of AF which might be enriched. Thus far, media containing rumen fluid from cattle and sheep were often used for the isolation of AF from non-rumen habitats, but this does not reflect the conditions of the isolation environments [19]. A potential solution would be the cultivation of AF in a medium devoid of rumen fluid, as recommended by Lowe et al., as it did not affect AF growth compared to AF growth in RF medium [26]. Some media without rumen fluid were adapted from the isolation and enumeration of anaerobic bacteria from non-rumen habitats [27,28]. However, the currently used standard AF medium has also been derived from the media used for cultivation of rumen bacteria [22,29,30].

Many representatives of the known genera were isolated and described in the past few years, proclaiming the exploration of different habitats, optimization, and variation in the isolation methodologies. As recently reported, strains of the genus *Aestipascuomyces* were isolated after storing the sample in a freezer (−20 °C) for two years [31]. Similarly, strains of the genus *Liebetanzomyces* were isolated after a prolonged incubation of 15 days instead of 3–4 days in the roll bottle [12]. Slight modifications in the isolation technique have been witnessed to give access to previously unidentified genera.

Most often, the diversity of AF in non-rumen habitats has been explored using rumen fluid-based media. However, this introduced bias may affect the diversity within enrichments and the further the strains that can be isolated. Therefore, it is hypothesized that the adjustment of suitable media to the conditions in the examined habitats should thus increase the chance for the successful enrichment and may better reflect the true diversity. Hence, in this study, we compare enriched cultures in three different media compositions with a special focus on rumen fluid replacement with potential application in the future isolation of novel AF. To attempt this, we studied for the first time the diversity of AF enriched from the feces of ruminant foregut fermenter (yak), pseudo-ruminant foregut fermenters (llama and guanaco), and hindgut fermenters (horse and Asian elephant) in three different media, containing either rumen fluid (RF), depleted rumen fluid (DRF) or no rumen fluid (NRF) without and with vitamins (NRF_V). The results should illustrate the role of rumen fluid in the medium for AF isolation from non-rumen habitats. To expand the cultivation-based observations, a specific primer set for *Neocallimastigomycetes* was used in this study to examine the diversity of AF in the original fecal samples and enrichment cultures. Our hypothesis was that a medium simulating the rumen environment (RF medium) is not suitable for the enrichment of representative AF genera from non-rumen samples, thus, capturing only part of the AF diversity present, and might even hamper the enrichment of important AF genera. The DRF medium tested in this study was expected to support the growth of fastidious AF without supporting the growth of undesired bacterial growth and can be used to complement the arsenal of commonly applied media. Further, we expected that a higher diversity of AF can be harvested from non-rumen samples employing NRF medium.

## 2. Materials and Methods

### 2.1. Sample Collection

Fresh fecal samples were collected from two groups of Przewalski’s horses (*Equus ferus przewalskii*) from the Wildnispark (Zurich, Switzerland) in August 2020. One group comprised grass grazing and roughage-fed sterilized males (horse group 1), and the other group, which comprised grass grazing females and one male, were additionally fed with multigrain straw pellets with roughage (horse group 2). Fecal samples from Asian elephant (*Elephas maximus*), llama (*Lama glama*), guanaco (*Lama guanicoe*), and yak (*Bos grunniens*) were collected from the Zoo Zurich (Switzerland) in May 2021. For each animal group, at least three or more droppings were collected from three to seven individuals. Sampling bags were tightly packed and excess air was removed before closing the bags to ensure a relatively oxygen-free environment [14,32]. The samples were brought to the lab within one hour and used to inoculate three different media. Left over samples were stored at −20 °C until DNA extraction was performed (see Section 2.3).

### 2.2. Media Preparation and Enrichment of AF

All media bottles were prepared under pure CO_2_ gas phase, using the anaerobic techniques described by Miller and Wolin [33]. One liter of RF medium consisted of 3 g yeast extract, 10 g tryptone, 150 mL of solution 1 (gL^−1^) [3 K_2_HPO_4_], 150 mL of solution 2 (gL^−1^) [3 KH_2_PO_4_, 6 (NH_4_)_2_SO_4_, 6 NaCl, 0.6 MgSO_4_·7H_2_O and 0.6 CaCl_2_·2H_2_O], 150 mL of clarified rumen fluid, 10 mL of trace element solution (gL^−1^) [0.25 MnCl_2_·4H_2_O, 0.25 NiCl_2_·6H_2_O, 0.28 Na_2_MoO_4_·2H_2_O, 0.2 FeSO_4_·7H_2_O, 0.05 CoCl_2_·6H_2_O, 0.04 Na_2_SeO_3_, 0.03 NaVO_3_, 0.026 ZnSO_4_·7H_2_O, 0.021 CuSO_4_, in 0.2 M HCl solution], 1 mL resazurin (0.1%), 1 mL hemin solution (0.05%), 6 g NaHCO_3_, 1 g L-cysteine-HCl, and 0.35 g of milled wheat straw (≈2 mm particle size) as a carbon source. For DRF and NRF media, 150 mL of rumen fluid was replaced with DRF or distilled water, respectively. The DRF was prepared according to the method described by Leedle et al. [34] (termed as incubated clarified rumen fluid), by supplementing salt solution one and two in fresh non-sterile rumen fluid, incubated for five days at 39 °C under oxygen-free atmosphere, and clarified by centrifugation at 10,000× *g* for 15 min before use. The prolonged incubation of five days allowed rumen bacteria to ferment easily degradable carbohydrates present in the rumen fluid.

A parallel experiment with an addition of 0.1% vitamin solution 1 (gL^−1^) [0.01 Thiamine HCl, 0.2 Riboflavin, 0.6 Calcium pantothenate, 0.6 Niacin, 1 Nicotinamide, 0.05 Folic acid, 0.02 Cyanocobalamin, 0.2 Biotin, 0.1 Pyridoxamine, 0.1 p-Aminobenzoic acid] [24] to NRF medium (NRF_V) was performed only for samples from the Zoo Zurich. The addition of vitamin solution was attempted to attain the micronutrient requirement of AF in NRF medium. This approach was added during the second sampling, where horse samples were not processed due to the time lag in the sample collection and experimental setup. Rumen fluid was collected from a healthy fistulated cow at AgroVet-Strickhof, Switzerland, and was clarified by centrifugation twice at 10,000× *g* for 15 min before use.

For inoculation, about 5 g of each fecal sample was added to 45 mL of sterile NRF medium, and then serially diluted up to 1:100 in NRF, RF, and DRF medium (*n* = 1), with 0.1% *v/v* penicillin G sodium salt, streptomycin sulphate, and ampicillin sodium salt (200 µg, 200 µg, and 100 µg/mL final concentration) added to restrict bacterial growth. All bottles, enrichment cultures, and the bottles with fecal samples were incubated stationary at 39 °C for 5 days in the dark. After five days of incubation, a visual assessment for AF growth (i.e., floating mat of straw and/or clumping of the straw), and a microscopic examination was performed. Biomass from the AF positive bottles was then centrifuged at 13,000× *g* for 10 min in sterile 50 mL falcon tubes and the obtained pellet was used for DNA extraction. The *Neocallimastix frontalis* strain GBF20D4 cultivated in all three media was used as a positive control in this study. The strain was isolated at ZHAW from Chamois (*Rupicapra rupicapra*) feces using DRF medium [35]. Negative controls were composed of uninoculated media bottles processed under the same conditions.

### 2.3. DNA Extraction, Library Construction, and Illumina Sequencing

Prior to DNA extraction, samples were washed using the method modified from [36] in sterile 100 mM PBS with 0.85% KCl, and then in sterile PBS in order to remove inhibitors. A total of 200 mg of washed sample was used for the total genomic DNA extraction using a GenElute Soil DNA Isolation Kit (Sigma, St. Louis, MO, USA), following the manufacturer’s instructions. The polymerase chain reactions (PCR) were performed using the AGF-LSU-EnvS primer pair amplifying the D2 LSU region according to Young et al. [8]. The generated amplicons, along with the attached forward Illumina adapter: 5′-TCGTCGGCAGCGTCAGATGTGTATAAGAGACAG-3′ (33 bp) and a reverse Illumina adapter: 5′-GTCTCGTGGGCTCGGAGATGTGTATAAGAGACAG-3′ (34 bp) were sent for Miseq Illumina sequencing (2 × 300 bp chemistry) to an external service provided by the Core Facility Microbiome at ZIEL—Institute for Food & Health, Technical University of Munich, Germany. The amplicon sequences were processed in QIIME2, as described in Section 2.4.

### 2.4. QIIME2 Pipeline

QIIME2 [37] was used to analyze and visualize the amplicon sequencing datasets. All raw amplicon sequencing datasets were imported into the QIIME2 platform and were demultiplexed into individual samples based on the identification barcode sequence. The demultiplexed datasets were then denoised using the DADA2 [38] implementation in the QIIME2 pipeline. In the denoising step, paired-end amplicon sequences were filtered, trimmed, and merged into unique sequences, i.e., amplicon sequence variants (ASVs). In the denoising process, DADA2 also tried to remove chimeric sequences using a de novo-based approach. The de novo-based chimera detection utilized high abundance ASVs as reference sequences to probe for hybrid sequences that were potentially generated during the PCR step. On top of DADA2, VSEARCH [39] was used with more strict parameters for denoising (--p-trim-left-f 15 --p-trim-left-r 15 --p-trunc-len-f 240 --p-trunc-len-r 160 --p-min-fold-parent-over-abundance 4) and Uchime de novo (--p-mindiv 0.2 --p-minh 0.05) to further remove chimera sequences. Finally, the high quality of the sequences was ensured by manual checking, and filtered reads were used for further analysis. For alpha diversity, the Shannon diversity index was determined as a quantitative measure of community richness, and the Jaccard distances were determined as qualitative measure of the community dissimilarity in beta diversity using the q2-diversity plugin of QIIME2. Additionally, principal coordinates analysis (PCoA) was performed to compress the information of Jaccard distances to a lower dimensionality for visualization.

In addition to analysis in QIIME2, phylogenetic analysis was performed using MEGA11 [40]. The reference alignment contained sequences of representatives of the 20 characterized genera of AF downloaded from NCBI GenBank and novel clades of uncultured sequences from Hanafy et al. [7]. ASVs with an abundance of more than 0.25% in each animal were considered to be authentic and included in the phylogenetic analysis [41]. The sequences were then aligned and trimmed for the D2 region (approx. 350 bp). The aligned sequences were used to construct a phylogenetic tree in MEGA11 using the Neighbor Joining method with the Tamura-3 parameter model, and the stability of the nodes was tested by 1000 bootstrap replications. *Chytriomyces* sp. WB235A (DQ536593) was used as an outgroup. Taxonomic classifications were carried out using the native implementation of the QIIME2 pipeline called q2-feature-classifier. Since there is a significant lack of reference sequences for AF, we compiled a robust in-house 28S rDNA (LSU) sequence database of AF [8]. These LSU reference sequences include novel AF species and genera that are published by Young et al. [8]. Using this customized reference database, a high-resolution classifier model was trained for taxonomic classification. In addition to the QIIME2 classifier, BLAST [42] was used to map particular unassigned ASVs to the customized reference sequences to further improve the phylogenetic classification. Based on the results from the taxonomic analysis, ASV reads were clustered at the genus level and relative abundances were calculated from the absolute abundance per sample. GraphPad Prism 9.4.0 was used to visualize a heatmap of relative abundance.

The raw sequence data were submitted to NCBI under the BioProject-SUB11496540. Generated ASVs were deposited under the accession numbers ON819032-ON819177.

## 3. Results

### 3.1. AF Enrichment Cultures in Different Media

Visual observations and microscopic examinations of all samples provided sufficient information to judge if AF were growing. AF growth was observed in most of the samples in all three media, except for horse group 2, where no growth was observed in DRF and NRF media. This, however, was most likely due to oxygen exposure caused by leaky rubber stoppers (Table S1). Acceptable AF growth with little bacterial contamination was observed more often in higher dilutions, hence, the third dilution (10^−3^) was chosen for amplicon sequence analysis (Table S1). In the higher dilutions of NRF medium (10^−3^), bacterial contamination was less often observed compared to RF and DRF media. In the enrichment cultures in NRF medium obtained from llama and elephant feces, AF growth was not noticed by visual inspection, but a variety of filamentous growth was observed during microscopic examination.

### 3.2. Sequencing Results and Diversity Analysis

The recently developed *Neocallimastigomycetes*-specific primer pair [8] used in this study generated 775,703 reads—76% of filtered reads were identified as unique ASVs (Table S2). The majority of ASVs were affiliated with *Khoyollomyces ramosus* (28.9%), *Neocallimastix* spp. (23.1%), *Piromyces* spp. (14.0%), *Anaeromyces* spp. (13.5%), and *Caecomyces* spp. (9.4%), whereas the remaining 6.5% were identified as potential novel clades (Table S3).

**Figure 1:**
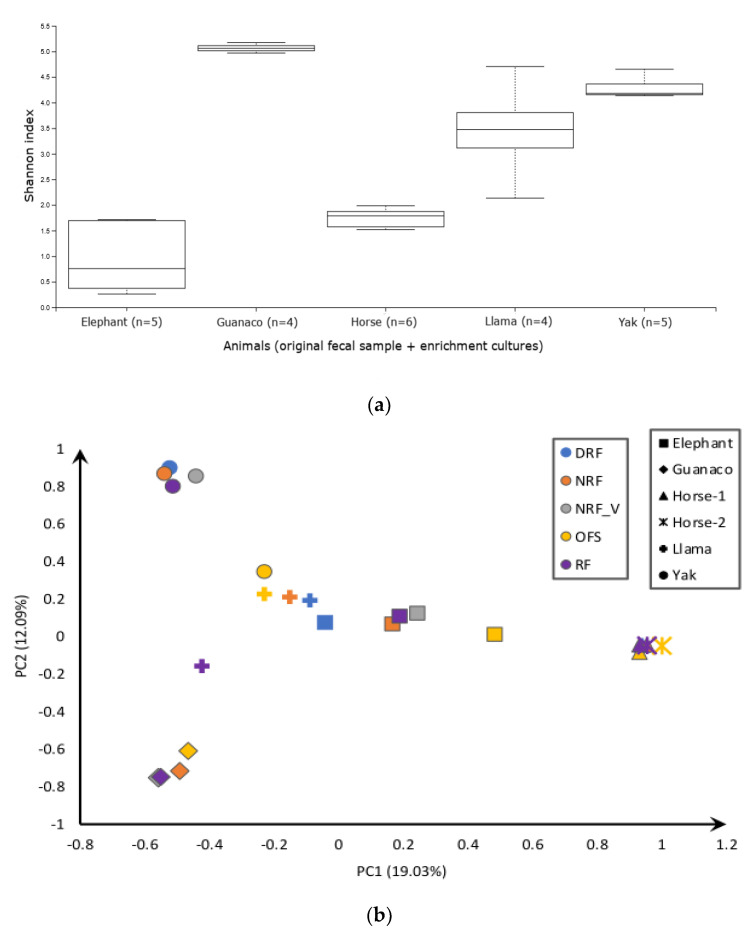
(**a**) Comparison of the alpha diversity (Shannon’s diversity index) among different groups of animal fecal samples (OFS) and enrichment cultures; (**b**) principal coordinates analysis (PCoA) plot of the beta diversity calculated using the Jaccard distance values of all original fecal samples (OFS) and enrichment cultures in the different media where RF, DRF, NRF, and NRF_V represent the media with rumen fluid, depleted rumen fluid, no rumen fluid, and no rumen fluid with vitamins, respectively.

**Figure 2:**
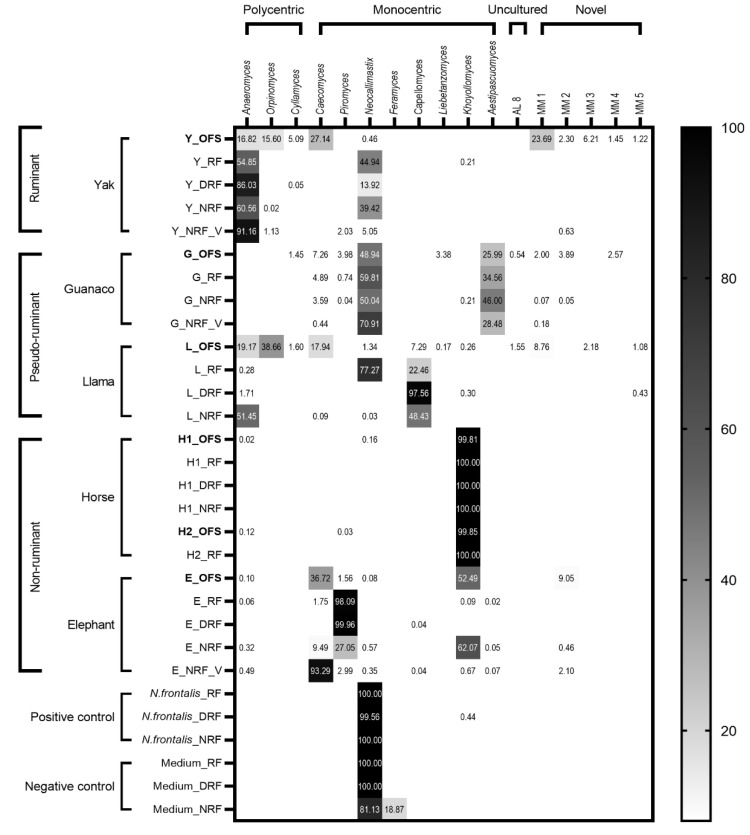
Heatmap showing the relative abundance of ASV reads at genus level from various original fecal samples (in bold) and respective enrichment cultures along with positive and negative controls. The details of the genera listed on top encompassing more than one species are listed in Table S3.

*K. ramosus* ASVs were abundant in only two animals, Przewalski’s horse and elephant. These samples also shared the highest number of reads but were the least diverse as shown in the alpha diversity plot (Figure 1a and Table S3). The alpha diversity (Figure 1a) clearly indicated a high species richness from guanaco, yak, and llama samples. It encompasses multiple genera, mainly *Neocallimastix* spp., *Orpinomyces* spp., *Anaeromyces* spp., *Caecomyces* spp., and *Aestipascuomyces dupliciliberans*, but shared a relatively low number of reads of the total amplicons (Figure 2).

**Figure 3:**
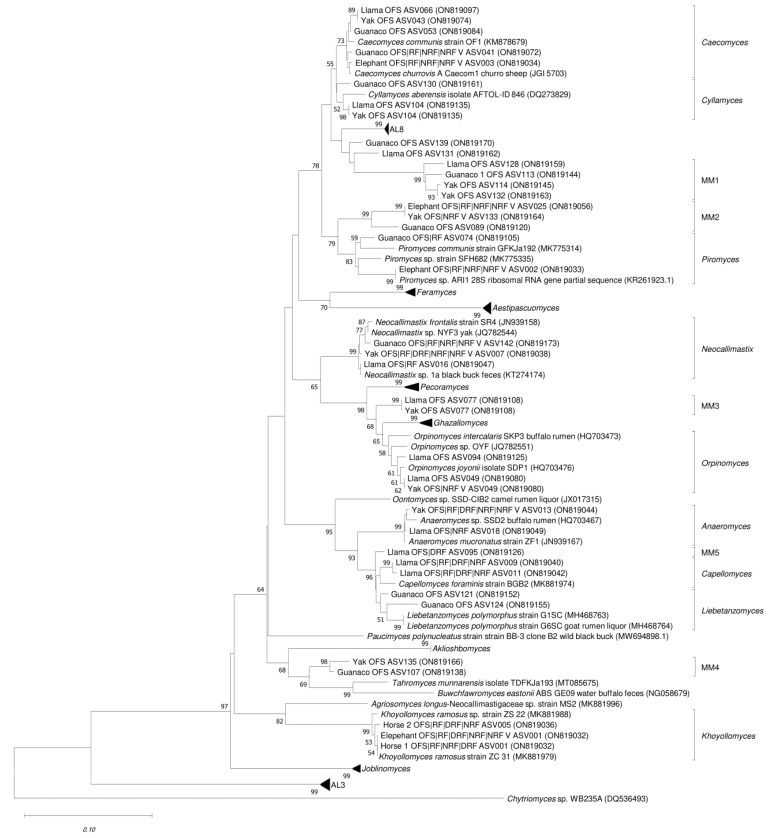
Phylogenetic tree of the D2 region of 28S rRNA gene sequences. Reference sequences from characterized AF isolates were included with the NCBI GenBank accession number. Previously described sequences of uncultured anaerobic fungi were taken from Hanafy et al. [7]. Sequences obtained in this study are defined with their respective ASV number along with NCBI GenBank accession number and assigned to the respective genera or novel clades (MM1 to MM5) with identification brackets. Using MEGA 11, sequences were aligned using Clustal W, and a Neighbor Joining Tree was constructed with a default setting of the Tamura 3-parameter model. Bootstrap values are based on 1000 replicates, and only values with >50% bootstrap support are shown. The tree was rooted with *Chytriomyces* sp. WB235A.

The original fecal sample (OFS) of yak (as rumen positive control of this study) harbored multiple genera including sequences affiliated with *C. communis* var. *churrovis*, *Anaeromyces mucronatus*, *Orpinomyces* spp., and some unidentified lineages (Figure 2 and Figure 3). Conversely, enrichment cultures in three applied media yielded only *Neocallimastix frontalis* and *A. mucronatus* sequences. In yak enrichment cultures, very little difference in diversity was observed between three media (Figure 1b), except in NRF_V enrichment culture, which displayed *Orpinomyces joyonii* and *Piromyces* spp. additionally (Figure 2). Similarly, the guanaco sample also showed high richness with most of the sequences falling into the genera *N. frontalis* and *A. dupliciliberans*, and some ASVs into *Caecomyces communis* var. *churrovis*, *Piromyces* spp., and novel genera (Figure 2 and Figure 3). The RF and NRF enrichments of guanaco samples displayed comparable diversity and abundance by favoring *N. frontalis*, *A. dupliciliberans*, and *C. communis* var. *churrovis*, whereas no growth was observed in DRF medium (Table S1). Llama OFS exhibited sequence assignments to *Orpinomyces* spp., *A. mucronatus*, and *C. communis* var. *churrovis*, resembling the diversity of the yak fecal sample (Figure 2). The similarity of the llama and yak fecal samples can also be seen in the beta diversity plot (Figure 1b). Surprisingly, RF medium enriched mostly *N. frontalis-* and *Capellomyces foraminis*-related sequences from the llama OFS, while NRF medium favored the growth of *A. mucronatus* and *C. foraminis*, and DRF medium favored mostly *C. foraminis*.

Compared to yak, guanaco, and llama samples, elephant OFS showed lower diversity, with *K. ramosus* and *C. communis* var. *churrovis* and the novel clade MM2 (minimal medium) being the most abundant representatives (Figure 2). Here, RF and DRF mostly enhanced the *Piromyces* sp.-related sequences that were identified as least abundant sequences in the OFS sample. On the contrary, *K. ramosus*, *Piromyces* spp., *C. communis* var. *churrovis*, and some MM2 ASVs were observed in the NRF enrichment culture, mimicking the results of the original fecal sample. Surprisingly, NRF enrichment culture—with the addition of the vitamin solution—specifically favored the detection of *C. communis* var. *churrovis*-related sequences (Figure 2). In horse samples, *K. ramosus* showed the highest abundance of sequences, regardless of the isolation medium. In group 1 of horse samples, all the three tested media yielded *K. ramosus* sequences, with the highest abundance found in NRF medium. In group 2, RF medium showed an equal number of *K. ramosus* reads, as did the original fecal sample (Figure 2).

The positive control (*N. frontalis* pure culture) of this study exhibited more *N. frontalis* sequences in RF medium than in DRF and NRF media (Table S3). The negative media control contained 4855 and 1765 *N. frontalis* reads in RF and DRF media, respectively, and 86 *N. frontalis* and 20 *Feramyces austinii* sequences in the NRF medium.

### 3.3. Phylogenetic Assignment

Sequences from fecal samples and enrichment cultures were assigned to 10 known genera in the Neighbor Joining Tree (Figure 3), including the polycentric genera *Cyllamyces aberensis*, *Orpinomyces* spp., *Anaeromyces* spp., and the monocentric genera *Caecomyces* spp., *Khoyollomyces ramosus*, *Piromyces* spp., *Neocallimastix* spp., *Capellomyces foraminis*, *Liebetanzomyces* spp., and *Aestipascuomyces dupliciliberans*. Some sequences were not assigned to any of the 20 previously described genera and formed new lineages designated as MM1 to MM5 (minimal medium). The sequences from these novel clades did not show any similarity to previously described groups (AL3–AL8) that were defined with an ITS1 marker and were later confirmed using the ITS1 and LSU D1/D2 region [7]. The D2 region of these novel groups showed significant divergence and were placed apart from the currently known genera of AF. Several ASVs from guanaco, yak, and llama were clustered in the clade MM1, rooting in the polyphyletic clade AL8. Some sequences (ASV139 and ASV131) from guanaco and yak were assigned to clade AL8 but with low bootstrap support (<50%) and low sequence identity. Similarly, several sequences from guanaco OFS, elephant OFS, and NRF and NRF_V enrichment culture from elephant were placed in the monophyletic clade MM2 close to the genus *Piromyces* and showed 89.9% sequence identity with *Piromyces communis* strain GFKJa192 (MK775314). The clade MM3 rooted between *Pecoramyces* and *Ghazallomyces* contained sequences from llama and yak feces. Group MM4 was rooted in the *Paucimyces*-*Aklioshbomyces*-*Tahromyces*-*Buwchfawromyces* clade and contained OFS sequences from yak and guanaco. ASV095 (ON819126) from llama feces and DRF enrichment were grouped to MM5 as a sister branch of *Capellomyces* and *Liebetanzomyces.*

Putative novel sequences encountered in guanaco OFS (ASV130, Table S3) showed 97.3% sequence identity with *Cyllamyces aberensis* (DQ2738329) and were assigned to the genus *Cyllamyces*. Abundant sequences ASV009 and ASV011 (Table S3) from llama enrichment cultures in different media were assigned to a sister clade with the genus *Capellomyces*, showing 97.9% sequence identity with *Capellomyces foraminis* strain BGB2 (MK881974). Sequences affiliating with the novel clades MM1 to MM5 were also encountered in the study of Young et al. [8], where these clades were named *Neocallimastigaceae* clade YL1, cand. *Piromyces potentiae* (sp. 8), *Orpinomyces* III, *Neocallimastigaceae* clade NC3 and *Neocallimastigaceae* clade YL3C, respectively.

## 4. Discussion

This study is the first attempt in comparing three different media to determine the most suitable medium to capture the maximum of the natural diversity of AF residing in a ruminant (yak), two pseudo-ruminant foregut fermenters (llama and guanaco), and two hindgut fermenters (elephant and Przewalski’s horse). In the presented comparison of three different media, a particular focus was put on the usage of clarified rumen fluid, which is commonly used for AF isolation and cultivation. The use of rumen fluid was initially intended to simulate the rumen conditions in order to obtain the maximum diversity of AF from rumen habitats [3,11]. Regular cultivation of AF is often practiced in medium containing rumen fluid which is usually obtained from bovine animals, even if it might not be ideal for the cultivation of AF from non-rumen hosts and might misleadingly influence the estimation of the AF diversity in such habitats [10,19]. Media lacking rumen fluid were already proven to equally support the growth of *N. frontalis* and *Piromyces* spp. [24,26], and thus, might be the better alternative to examine non-rumen habitats.

### 4.1. Diversity of AF in Rumen and Non-Rumen Origin Samples

More sequences were obtained from fecal samples of hindgut animals (i.e., horse and elephant) than the other samples, but they also exhibited the least diversity (Table S3). This corroborated the results of Liggenstoffer et al. [15], who also obtained more sequences from hindgut and pseudo-ruminants than ruminants sampled at the Oklahoma City Zoo. This correlation of relative AF quantification in different stomach types along with diversity should be further investigated. Surprisingly, the different diet of two sampled Przewalski’s horse groups did not affect the AF diversity as observed in previous studies, where a shift in AF community was reported for goats fed on a high grain diet [43]. A possible reason could be the host specificity of genus *K. ramosus*, previously described as clade AL1, which has been reported to occur in many hindgut and foregut fermenters [7,15] and is also known to be a prominent member of different parts of the equine gut [44]. In this study, sequences belonging to *K. ramosus* were, for the first time, also identified in elephant samples (Figure 2). A recently published article conducted on African elephants demonstrated the presence of *Piromyces* and *Pecoramyces* with two novel clades, supporting the isolation of *Piromyces* spp. from elephant samples in this study [6,10]. Contrasting results observed by Nicholas et al. [45] showed the occurrence of *Anaeromyces* in African elephants, which could again support the theory that the geographic distribution influences the composition of the AF community [6].

The AF from llama samples exhibited a unique mixture of sequences affiliated to polycentric and monocentric genera, similar to the data from Liggenstoffer et al. [15]. However, in the current study, *Orpinomyces joyonii* sequences were most abundant in the llama sample. Additionally, llama feces harbored sequences assigned to the novel groups MM1, MM3, MM5, and a putative new species of genus *Cyllamyces*. Guanaco, which belongs to the same family as llama (*Camelidae*), also showed high diversity, but mainly featured sequences related to monocentric AF genera (Figure 2). Rabee et al. reported that *N. frontalis* and *Cyllamyces* sp. strains were predominantly found in the gut of *Camelidae* [46]. In the current study, guanaco samples also showed a predominance of *N. frontalis* and a wide variety of novel sequences (MM1-2 and AL8).

The diversity in the yak sample exhibited a variety of filamentous polycentric, monocentric, and bulbous genera (Figure 2). Similar results were observed in a study on rumen samples from different fistulated and slaughtered wild yaks from China, where the presence of mainly *N. frontalis*, *O. joyonii*, and uncultured *Caecomyces* sequences was reported [18]. Four different cattle breeds—namely, gayals (*Bos frontalis*), yaks (*Bos grunniens*), and Yunnan and Tibetan yellow cattle (*Bos taurus*)—were shown to harbor *Piromyces*, *Anaeromyces*, *Cyllamyces*, *Neocallimastix*, and *Orpinomyces* [47]. In this study, we found the genus *Caecomyces communis* var. *churrovis* and the novel clade MM1 to be most abundant, followed by *Anaeromyces mucronatus* and *Orpinomyces* spp. sequences. Surprisingly, 34.8% of the total sequences in yak OFS were shared by novel lineages (MM1-5). A possible reason for the discovery of more novel genera in yak samples in this study compared to previous studies is the better coverage of AF diversity with the used specific primer pair. Such novel genera identified by sequencing can become a scope of future isolation surveys which apply a variety of cultivation media.

### 4.2. Influence of the Medium on the Observed Diversity of AF

The isolation medium plays a crucial role in enriching thus far uncultured species from environmental samples. This study clearly indicates that the diversity of AF during the isolation process is selectively influenced by the medium used. For example, in RF enrichment cultures of llama, most of the sequences were affiliated to *N. frontalis*, whereas *O. joyonii* and *A. mucronatus* were most abundant in the feces. Additionally, other studies on feces from *Camelidae*, specifically llama, mostly resulted in the isolation of *Neocallimastix* spp. [48,49,50]. The yak sample in this study also enriched *N. frontalis* in RF medium instead of *C. communis* var. *churrovis* and clade MM1 which are abundantly present in the feces. This was also seen in the isolation from the rumen of cattle from India, where *Anaeromyces* spp. and *Orpinomyces* spp. were predominantly present [51]. Additionally, the isolation from rumen of yak from China broadly showed the presence of *N. frontalis* [18]. RF and DRF media failed to enrich *K. ramosus* sequences, which were predominant in the elephant OFS, probably due to the adverse rumen fluid composition of the medium. Thus, isolation results can vary depending on the source and composition of the rumen fluid used, and on the diversity present in the host animal. In the current study, in the positive control bottles, more (49,001) *N. frontalis* ASV reads were obtained in RF medium compared to DRF (10,568) and NRF (4680; Table S3). This indicates the presence of *N. frontalis* in the fistulated cow (source of rumen fluid in this study). RF supplementation conditions possibly favored the growth of *N. frontalis* in enrichment cultures of yak and llama. Further, 4855 sequences of *N. frontalis* were found in the RF, 1765 in the DRF, and 86 in the NRF negative control. *N. frontalis* might thus have come from the rumen fluid even after autoclaving as residual DNA [22,32,52,53]. The *N. frontalis* and 20 *F. austinii* sequences found in the NRF negative control may come from well-to-well, background contamination, or barcode leakage [41,54]. Spurious contamination was also observed in the water sample (no template control) with a traceable amount of *K. ramosus* sequences (18 reads) obtained. However, these few sequences did not seem to hamper the results, as these genera were not detected throughout the samples.

This study is the first using depleted rumen fluid (DRF) for the enrichment of AF. It was used to simulate the natural environment but without free sugars present in rumen fluid [34,55,56], which was expected to result in less bacterial contamination in AF enrichment cultures. We found that DRF medium supported the growth of specific AF genera. Compared to RF medium, the change in diversity in DRF medium could likely be due to the change in rumen fluid composition during the depletion process. These differences were most noticeable in isolation trials from llama samples. For the enrichment trials from llama samples, DRF medium explicitly supported the growth of *C. foraminis,* while RF medium favored *N. frontalis* as a result of change in rumen fluid composition. Surprisingly, *Orpinomyces* spp., *A. mucronatus*, and *C. communis* var. *churrovis,* which were abundant in the llama feces, were not enriched in any of the applied media. Apparently, a different medium composition is needed to support the growth of these genera. In DRF enrichment cultures from horse, the medium change did not influence the diversity as the original sample was less diverse. However, the DRF enrichment culture from elephant revealed more sequences related to the genus *Piromyces* sp. compared to RF medium, which were the least abundant in the original sample. In guanaco samples, no valid conclusion could be drawn because of a lack of growth in DRF medium, since AF propagation was probably inhibited by the overgrowth of bacteria (Table S1). Bacterial contamination in AF enrichment cultures can be a problem hindering the growth of AF, as observed in the antagonistic relationship between the anaerobic rumen bacterium *Fibrobacter succinogenes* with *Anaeromyces robustus* and *C. communis* var. *churrovis* [57].

The cultivation of llama and elephant samples in NRF medium resulted in distinct diversity in the enrichment cultures, whereas equal results were obtained using NRF medium for guanaco, horse, and yak samples. In elephant samples, NRF cultivation, compared to RF and DRF media, produced a higher diversity and detection of more genera, including the novel sequences (MM2) in enrichments. Interestingly, the addition of the vitamin solution to the NRF medium favored the growth of *C. communis* var. *churrovis* and novel clade MM2, which illustrates how small changes in media composition can have a big influence on the isolation results. In llama samples, NRF medium favored *A. mucronatus* enrichment and a putative novel *Capellomyces* species, which was not found in RF enrichment culture.

Many expected AF genera detected in OFS were unable to grow in the tested media; this may be due to several possible factors—substantial bacterial contamination is amongst the most likely factors, possibly linked to antagonistic inhibition [57], which was more commonly observed with RF and DRF media (Table S1). This is not surprising, as the standard media for AF cultivation were derived from anaerobic rumen bacterial media [22]. Modifying the antibiotic cocktail to suppress the growth of multidrug-resistant bacteria may permit the growth of novel AF genera. AF cultivation does not require a complex media composition, as shown by the results obtained with NRF enrichment medium, and also by the findings of Lowe et al. [26] and Teunissen et al. [24]. Additionally, the use of yeast extract and tryptone in the medium is also debatable, as an omission of these ingredients did not restrict the growth of AF in substrate utilization studies for *Aestipascuomyces dupliciliberans* [58]. Hence, optimization of the isolation media for different habitats to study the diversity of cultivable AF is clearly useful. Importantly, other factors such as salt solution, the buffer system, a suitable temperature, and the pH value should also be optimized according to the conditions in the sampling source to isolate—as best as possible—the natural diversity from various habitats.

## 5. Conclusions

This study found that the use of rumen fluid in the medium has a major impact on the growth of anaerobic fungi (AF) from different hosts and can lead to the selective enrichment of particular genera. A medium devoid of rumen fluid (NRF) yielded an equal or a higher diversity than rumen fluid-based media, indicating the potential utility of such media to enrich hitherto uncultured AF. Slight variations to the medium composition, such as the addition of the vitamin solution, specifically altered the obtained diversity in enrichment cultures. These findings support our hypothesis that the use of cow rumen fluid to simulate the gut content is not necessary if AF are to be enriched and isolated from different hosts. Adding digesta fluid from the same host as the sampling source in the growth medium would be a more realistic environment simulation for prospective AF isolation. Additionally, isolation medium optimization should be re-addressed by considering the conditions in different habitats to more adequately study the diversity of cultivable AF.

## Data Availability

The raw sequence data were submitted to NCBI under the BioProject-SUB11496540. Generated ASVs were deposited under the accession numbers ON819032-ON819177. Reference sequences for AF, we have compiled a robust in-house 28S rDNA (LSU) sequence database of AF (reference sequences). These LSU reference sequences include novel anaerobic fungal species that are available under the publication of Young et al. [8].

## Supplementary Materials

The following supporting information can be downloaded at: https://www.mdpi.com/article/10.3390/microorganisms10101972/s1; Table S1: Visual observations in terms of AF growth, bacterial contamination, and medium condition in different media enrichments in three dilutions; Table S2: Results of raw data from fecal samples and enrichments after filtering through QIIME2 and Uchime pipeline; Table S3: Abundance of ASV reads in original fecal samples, enrichment cultures, and experimental controls.
